# Serum vitamin D levels can be predictive of psoriasis flares up after COVID-19 vaccination: a retrospective case control study

**DOI:** 10.3389/fmed.2023.1203426

**Published:** 2023-05-25

**Authors:** Emmanouil Karampinis, George Goudouras, Niki Ntavari, Dimitrios Petrou Bogdanos, Angeliki-Victoria Roussaki-Schulze, Efterpi Zafiriou

**Affiliations:** ^1^Department of Dermatology, University Hospital of Larissa, University of Thessaly, Larissa, Greece; ^2^Department of Rheumatology and Clinical Immunology, University Hospital of Larissa, University of Thessaly, Larissa, Greece

**Keywords:** psoriasis, SARS-CoV-2 vaccination, COVID-19, vaccine, disease exacerbation, vitamin D

## Abstract

**Introduction:**

Many patients with chronic inflammatory dermatosis such as psoriasis usually ask about the safety of COVID-19 vaccination and if it would affect the course of their disease. Indeed, many case reports, case series and clinical studies, reporting psoriasis exacerbation following vaccination against COVID-19, were published during the pandemic. Also, many questions arise regarding the existence of exacerbating factors of these flare ups, including environmental triggers such as the insufficiency of vitamin D levels.

**Methods:**

This is a retrospective study that measures alterations in psoriasis activity and severity index (PASI) not exceeding 2 weeks after the first and second dose of COVID-19 vaccinations in the reported cases and assesses whether such changes have any association with patients’ vitamin D levels. We retrospectively reviewed the case records of all patients with a documented flare up after COVID-19 vaccination in our department as well as those who did not, during a year.

**Results:**

Among them, we found 40 psoriasis patients that had reported vitamin D levels in the form of 25-hydroxy-vitamin D within 3 weeks after vaccination, including 23 with exacerbation and 17 without exacerbation. Performing χ^2^ and *t*-test controls for psoriasis patients with and without flare-ups, a statistically significant dependence emerged in the seasons of summer [χ^2^(1) = 5.507, *p* = 0.019], spring [χ^2^(1) = 11.429, *p* = 0.001] and in the categories of vitamin D [χ^2^(2) = 7.932, *p* = 0.019], while the mean value of vitamin D for psoriasis patients who did not have exacerbation (31.14 ± 6.67 ng/mL) is statistically higher [*t*(38) = 3.655, *p* = 0.001] than the corresponding value of psoriasis patients who had an exacerbation (23.43 ± 6.49 ng/mL).

**Discussion:**

This study indicates that psoriasis patients with insufficient (21–29 ng/mL) or inadequate (<20 ng/mL) levels of vitamin D are more prone to postvaccination aggravation of the disease while vaccination in summer, a period with the most extent photo-exposition, can be a protective factor.

## 1. Introduction

During the SARS-CoV-2 pandemic, dermatology and dermatologic care faced many challenges. To prevent infection with COVID-19, the two first mRNA vaccines were developed by Pfizer-BioNTech and Moderna. The Pfizer-BioNTech vaccine (BNT162b2) required two doses taken 21 days apart and provided 95% protection against COVID-19, while the Moderna vaccine (mRNA-1273) required two doses taken 28 days apart and provided 94.5% protection. Therefore, vaccination is strongly recommended to all individuals who do not have contraindications, including patients with psoriasis disease (1). In the year COVID-19 pandemic begun, there were 4,622,594 incident cases of psoriasis (2), who due to increased airway inflammation (3) and their immunosuppressive treatment were expected to have a worse COVID-19 clinical outcome. Consequently, protection of this category of patients against COVID-19 by vaccination was important. However, an increasing number of studies, are published reporting patients with psoriasis flare ups, ranging from case reports (4–6) to case report series (7–10). As per the analysis conducted by Wu et al. (11), the administration of the second dose was frequently linked to the worsening of the disease, with the time of occurrence ranging from 2 to 21 days in the new-onset group and from 1 to 90 days in the flare group.

Vitamin D is a type of fat-soluble steroid pre-hormone that is primarily synthesized in the skin through exposure to sunlight or can be acquired through diet. The most stable form of vitamin D is 25-hydroxy vitamin D [25 (OH)D], which has a half-life of 2–3 weeks and is widely used as an indicator of vitamin D levels in the body (12). The anti-inflammatory properties of vitamin D have a significant protective impact on the development of psoriasis by reducing inflammation in human monocytes and macrophages. In addition, it can decrease the production of various pro-inflammatory cytokines (13), which have connected psoriasis with chronic pruritus (14, 15), depression and anxiety (16). Vitamin D is essential in regulating the balance between the innate and adaptive immune systems and can lead to a decrease in autoimmune disease activity. It plays a critical role in maintaining normal immune function, and a deficiency can impede the body’s ability to keep the skin healthy, potentially leading to an increase in psoriasis flares (17). Also, vitamin D contributes to the cutaneous barrier’s integrity by controlling keratinocyte turnover, while vitamin D active metabolite, 1,25-dihydroxyvitamin D (1,25 (OH)_2_D), induces the production of antimicrobial peptides that have immune regulatory properties (18). The above-mentioned mechanisms indicate the interrelation between vitamin D levels and psoriasis while studies connect vitamin D lower levels with higher PASI scores and higher disease duration (19).

In addition to psoriasis, vitamin D seems to be correlated inversely to the clinical image of many dermatoses especially immune mediated skin disorders. Patients with hidradenitis suppurativa and vitamin D deficiency tend to have higher disease severity score and systemic inflammation levels, as evidenced by elevated serum CRP levels (20). Lower serum 25 (OH) D is associated with a more severe clinical presentation of atopic dermatitis (21) and scalp seborrheic dermatitis (22). Therefore, patients with inflammatory dermatosis and low vitamin D levels, when exposed to a potential trigger, are more prone to a disease exacerbation, which in turn results in a worse clinical image, as vitamin D protects against skin and systemic inflammation (20).

The objective of our retrospective study is to assess potential protective factors against psoriasis flare-ups following COVID-19 vaccination, including the status of vitamin D. Our findings may encourage additional multicenter research that could be advantageous for many patients with skin conditions in the future. For instance, if a protective relationship is discovered, our study may support the use of vitamin D supplements in psoriasis patients to prevent flare-ups after vaccination.

## 2. Study population and data collection

The study involved individuals with plaque psoriasis who visited the Psoriasis Department of the Dermatology Clinic at the University Hospital of Larissa between January 15, 2021 (around the commencement of public COVID-19 vaccination in Greece) and January 15, 2022.

To be eligible for the study, participants needed to have received one of the three most commonly administered COVID-19 vaccines in Greece, including BNT162b2 (Pfizer), AstraZeneca-Oxford, or Moderna, and have recorded PASI scores after both the first and second doses in their medical records. Additionally, participants needed to have reported their vitamin D levels as 25-hydroxy-vitamin D levels within 3 weeks following vaccination. The study did not have any age restrictions, but patients taking vitamin supplements or medications, or those with liver disease, kidney disease, malabsorption, inflammatory bowel disease, or any other illnesses that impact vitamin D levels in the blood were excluded. Patients who were obese or experienced excessive stress during the specified period were also excluded to minimize the impact of confounding factors such as stress that could further exacerbate the disease course.

The changes in psoriasis activity measured by the PASI score only included the period up to 2 weeks after vaccination. The PASI score evaluated the severity of psoriasis on each part of the body, such as the head, arms, trunk, and legs, by assessing the percentage of skin involvement and the severity of three clinical signs (erythema, induration, and desquamation) on a scale of 0 to 4. Only patients with recorded PASI scores after both the first and second vaccine doses were included in the study and those without recorded scores were excluded.

Serum 25-hydroxy-vitamin D levels were calculated by chemiluminescent immunoassay in our hospital biochemistry lab. The method used by the Elecsys Vitamin D total II assay involves the use of a capture protein that is labeled with a ruthenium complex, which is capable of binding 25 hydroxyvitamin D3 and 25 hydroxyvitamin D2. To prevent any interference from 24,25 dihydroxyvitamin D, a monoclonal antibody that is specific to this molecule is utilized (23). The categorization of the patients was done according to Endocrinology Society proposed Vitamin D status system, according to which, serum circulating level of 25 (OH) D < 20 ng/mL is considered deficiency while levels of 25 (OH) D of 21 ng/mL to 29 ng/mL indicate insufficiency and 30–100 ng/mL is the sufficiency range.

### 2.1. Statistical analyses

The Statistical Package for Social Sciences (SPSS) for Windows Microsoft corporation 2008 was used to perform the statistical analyses. The Chi-square test was used for discrete variables, and the independent *t*-test was used for continuous variables. Additionally, a logistic regression analysis was conducted with exacerbation as the dependent variable. Correlations were analyzed using Spearman’s correlation analysis. A result was considered statistically significant if *p* < 0.05.

## 3. Results

### 3.1. Descriptive characteristics of study population

**Figure 1:**
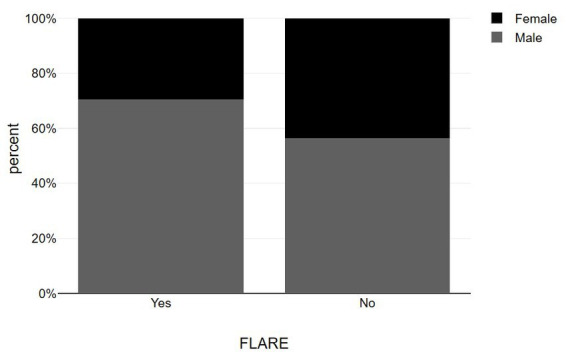
Bar graphs showing sex distribution among psoriasis patients with and without post-vaccination flare-up.

**Figure 2:**
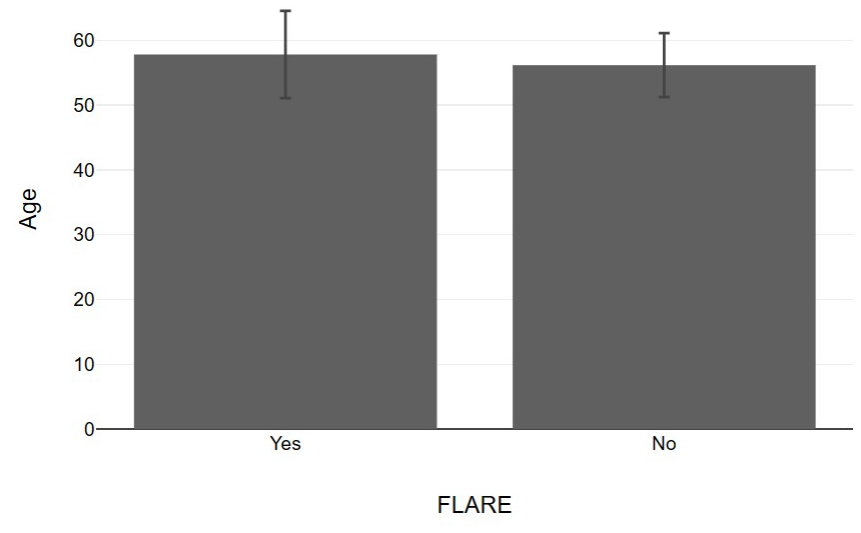
Bar graphs with 95% confidence interval showing age values between psoriasis patients with and without flare up.

Our study included 40 patients who had documented vitamin D levels and experienced psoriasis changes after receiving COVID-19 vaccines. Out of the 40 patients, 23 reported no change in their psoriasis score after the vaccination, while 17 experienced a flare-up and sought treatment at our department (Table 1). All patients had a pre-existing history of chronic psoriasis and had not previously been infected with COVID-19. There were no statistically significant differences in age and gender between the two groups (Figures 1, 2). Notably, all patients who experienced a flare-up had recorded vitamin D levels, as they underwent more comprehensive laboratory exams for further investigation. Most of the flares (88%) occurred after the second dose of the vaccine and 2/17 were AstraZeneca-Oxford vaccinations. Additionally, the severity of the flare-up in this group varied, ranging from less severe to erythrodermic pustular psoriasis (morphologic change). The PASI score of the flare group was 9.03 ± 3.7. The onset of the exacerbations occurred from 9 ± 2 days after vaccination and usually it was accompanied with extracutaneous manifestations in both categories ranged from local pain in the site of injection to arthralgia, fever, myalgias and somnolence. Among flare-up cases, there were 3 cases under topical treatment, 8 cases under biology therapy including etanercept (*n* = 1), methotrexate (*n* = 2), ustekinumab (*n* = 2), and secukinumab (*n* = 3) and 6 cases that withdrew treatment including methotrexate (*n* = 4), secukinumab (*n* = 1), and brodalumab (*n* = 1).

**Table 1:** The results of the chi-square and *t*-test analyses comparing psoriasis patients who experienced exacerbation after receiving COVID-19 vaccination to those who did not.

Variant	Psoriasis patients without exacerbation (57.5%, *N* = 23)	Psoriasis patients with exacerbation (42.5%, *N* = 17)	Statistics	*p*-value
Sex			χ^2^(1) = 0.825	0.364
Female	66.7% (*N* = 10)	33.3% (*N* = 5)		
Male	52.0% (*N* = 13)	48.0% (*N* = 12)		
Age	56 (±12.7)	58 (±14.2)	*t*(38) = −0.468	0.642
Season				
Summer	77.8% (*Ν* = 14)	22.2% (*Ν* = 4)	χ^2^(1) = 5.507	**0.019**
Spring	34.8% (*Ν* = 8)	65.2% (*Ν* = 15)	χ^2^(1) = 11.429	**0.001**
Winter	100% (*Ν* = 1)	0% (*Ν* = 0)	χ^2^(1) = 0.758	1.000+
Autumn	50% (*Ν* = 1)	50% (*Ν* = 1)	χ^2^(1) = 0.048	1.000+
Vitamin D levels	31.14 (±6.67)	23.43 (±6.49)	*t*(38) =3.655	**0.001**
Vitamin D categories			χ^2^(2) = 7.932	**0.019**
Deficiency	28.6% (*N* = 2)	71.4% (*N* = 5)		
Insufficiency	43.8% (*N* = 7)	56.3% (*N* = 9)		
Sufficiency	82.4% (*N* = 14)	17.6% (*N* = 3)		

+Exact Fisher value.*p* values < 0.05.

### 3.2. Psoriasis exacerbation and season of the year

**Figure 3:**
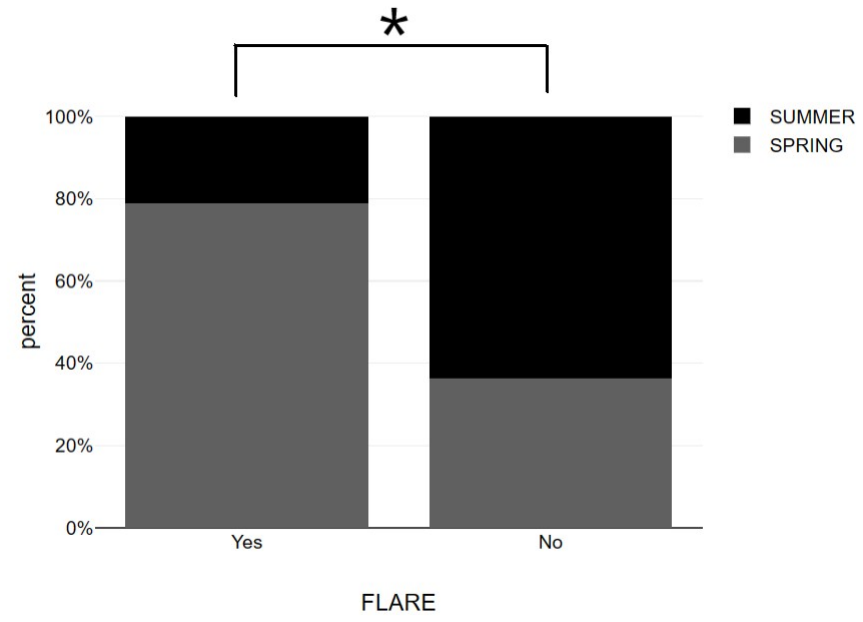
Bar graphs showing summer vs. spring distribution among psoriasis patients with and without post-vaccination flare-up. *p < 0.05, significantly different compared to flare up group.

Worth mentioning is that the most vaccinations occurred during the seasons of summer and spring according to the preventive measures against COVID-19 pandemic. Patients that participated in the study live in Larissa, a region of Greece, located at a latitude of 39.65 and a mean monthly duration of sunshine in this location varies from 98.8 h in December to 332.8 h in July (24). According to our results, psoriasis exacerbation after vaccination was negatively associated with summer [χ^2^(1) = 5.507, *p* = 0.019] while vaccination during spring was positively associated with disease flare up [χ^2^(1) = 11.429, *p* = 0.001]. Also, the incidence rates in the total sample were 42.5%. Among them, 22.2% occurred in summer and 65.2% occurred in spring (Figure 3 and Table 1).

### 3.3. Psoriasis exacerbation and vitamin D

**Figure 4:**
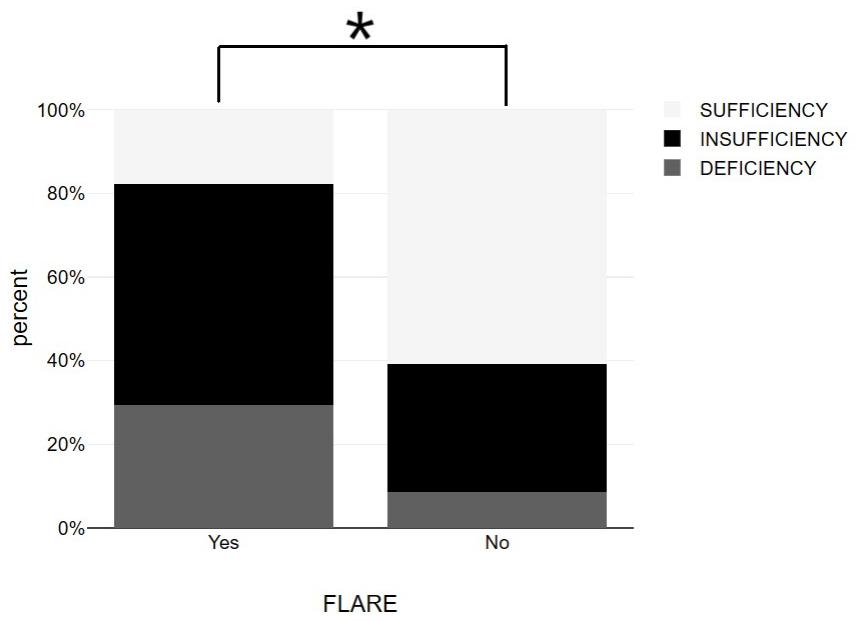
Bar graphs showing vitamin D status among psoriasis patients with and without post-vaccination flare-up. *p < 0.05, significantly different compared to flare up group.

**Figure 5:**
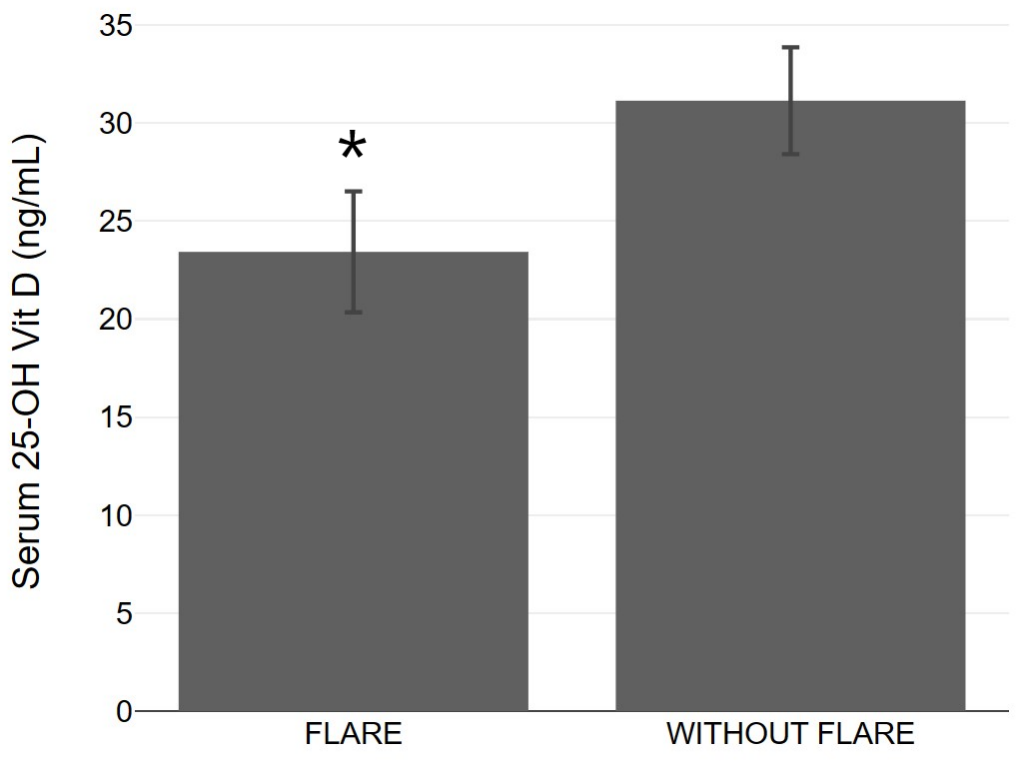
Bar graphs with 95% confidence interval showing serum 25-OH vitamin D between psoriasis patients with and without flare up. *p < 0.05, significantly different compared to flare up group.

Patients with psoriasis who did not experience exacerbation after vaccination had a statistically higher average vitamin D level (31.14 ± 6.67 ng/mL) compared to those who did experience exacerbation (23.43 ± 6.49 ng/mL) (Table 1 and Figure 1). The association between psoriasis exacerbation and vitamin D status was significant [χ^2^(2) = 7.932, *p* = 0.019]. Among all patients, 42.5% experienced flare-ups, while the percentage increased to 56.3% for patients with vitamin D insufficiency and 71.4% for patients with vitamin D deficiency (Figures 4, 5; Table 1). Although a negative correlation was observed between the PASI score of flare cases and their vitamin D levels, it was not statistically significant (*r* = −0.11, *p* = 0.674).

By using logistic regression model with psoriasis exacerbation as an independent variable, we found a statistically significant effect of the independent variable on the dependent variable (*p* < 0.001), with a high degree of adaptation (NagR2 = 0.699). Among the variables included in the model, only the “Vitamin D Values” variable was statistically significant (*p* = 0.034) and low levels of vitamin D were found to be the only predictor of a psoriasis flare-up (Figures 2–5).

## 4. Discussion

Vitamin D, also known as the “sunlight-hormone,” is recognized as an antioxidant and immunomodulatory agent, and is particularly important in the pathogenesis of psoriasis due to its association with low levels of 25 (OH) D and 1,25 (OH)_2_D in many psoriasis patients (13). This correlation may be explained by the tendency of some patients to cover their skin lesions and avoid sun exposure, which results in reduced vitamin D production (14). Studies have confirmed that vitamin D has the ability to increase the production of anti-inflammatory cytokines by reducing or blocking the production of pro-inflammatory cytokines like IL-6 and TNF-α, both of which play a part in the development of psoriatic skin. Consequently, vitamin D may play a significant role in the chronic autoimmune or inflammatory aspect of the disease (16). Also, proliferation and maturation of the keratinocytes seem to be promoted by low concentration of 25 (OH) D and inhibited at higher concentration (14). There is disagreement among studies regarding the effectiveness of oral vitamin D supplements in improving psoriatic lesions, with some studies reporting improvement and others reporting no improvement (25).

The relation between skin and UV radiation is complex, while the production of vitamin D is an important aspect; other directions need to be studied as far as psoriasis is concerned. Ultraviolet (UV) radiation alters the cytokine profile associated with psoriasis by redirecting the immune response away from the pro-inflammatory Th1/Th17 axis (26). The seasonal variation impact on psoriasis course is well established as most cases reported skin relief in summer and exacerbation in winter and few referred worsening of the skin disease due to photosensitivity (27).

Contrary to the beneficial role of UV and vitamin D, the immune response induced by the COVID-19 vaccine is reported to cause psoriasis exacerbation as well as new onset psoriasis. BNT162b2 vaccine is proven to stimulate Th1 cells, while in animal models it can lead to production of IL-17 (9). After receiving the AstraZeneca-Oxford COVID-19 vaccine, an increase in the production of tumor necrosis factor (TNF)-α and interferon (IFN)-γ by CD4+ T cells was observed (28). It is known that the IL-23/IL-17 axis is essential in the immunopathogenesis of psoriasis, with TNF-α and IFN-γ being the primary pro-inflammatory signals (29). The similarity in the cytokine profiles of psoriasis and the immune response induced by the COVID-19 vaccine could be the reason for the observed post-vaccination psoriasis flare-up.

The study conducted by McMahon et al. involved 414 participants who experienced skin issues after receiving anti-COVID-19 vaccines. The study reported a small number of flare-ups of chronic skin conditions, including psoriasis and atopic dermatitis (30). However, an increasing number of studies are published reporting patients with psoriasis flare ups, ranging from case reports to case report series. In those studies, COVID-19 vaccination information (vaccine type and dose), onset time of exacerbation after vaccination as well as clinical presentation and treatment status of the flare up cases presented differences.

Our study aligns with previous studies conducted by Sotiriou et al. (7) and Megna et al. (8) on psoriasis exacerbation following vaccination, which indicate that psoriasis worsening is mostly observed after the second vaccine dose and primarily presents as plaque psoriasis flare. Regarding the sex distribution of flare-ups, Megna et al. found a higher incidence among males and suggested male sex as a potential predictive risk factor, while our study did not find sex to be a significant contributor. Case series on the psoriasis flare have shown that BioNTech/Pfizer, Moderna and AstraZeneca vaccine can lead to psoriasis exacerbation. BioNTech/Pfizer vaccine reported more often than the other vaccines (11), a finding which is similar to our results. The onset time of a psoriasis flare ranged from 1 day to 90 days (11). However, we assessed psoriasis exacerbation within 2 weeks to avoid confounders. Post-vaccination onset of the exacerbation ranged from 5 to 32 and 2–25 days according to two case control studies that were performed in the same country as our study (10). Also, another important point is the definition of a psoriasis flare-up case. The study of Huang and Tsai (9) included only episodes of worsening of 50% of deterioration of PASI scores. Sotiriou et al. (7) and Megna et al. (8) reported a 9.8 ± 3.5 and 10.4 ± 4.7 PASI of the cases respectively, which is similar to the PASI range of our case report study (9.03 ± 3.7).

Similar to Megna et al. (8) we also observed that patients (8 out of 17) who were undergoing biological therapy reported a remission of their psoriasis. In our flare-up group, there were three patients that were following topical treatment and six patients that withdrew treatment-biology agent. The withdraw of immunosuppressive treatment before and after receiving the vaccine was a tactic which is used to improve the efficacy of the vaccination immunity response. Currently, patients with psoriasis are recommended to receive COVID-19 vaccinations while continuing their biological treatment, and some patients may need a three-dose schedule to achieve sufficient levels of neutralizing antibodies against SARS-CoV-2 (31). Studies have shown that the second dose of BNT162b2 vaccine can effectively produce antibody responses in patients who are taking methotrexate and targeted biologics, but a lower percentage of immunosuppressed patients may have detectable T-cell responses after the second dose (32).

Our study has some limitations. Firstly, the study population was small, and we did not consider variations in patients’ dietary habits that could have influenced the levels of 25 (OH)D. Moreover, due to the retrospective nature of the study, further research should be conducted to evaluate the effects of vitamin D or other protective agents against post-vaccination flare-ups of systemic skin diseases.

## 5. Conclusion

COVID-19 vaccination is strongly suggested to psoriasis patients. Our study is in line with previous studies that focused on psoriatic skin exacerbation after COVID-19 vaccination. Higher rates of exacerbation occurred in patients with low vitamin D while lower rates of psoriasis exacerbation were observed during summer—a season with extent sun-exposure.

## Data availability statement

The original contributions presented in the study are included in the article/supplementary material, further inquiries can be directed to the corresponding author.

## Ethics statement

The studies involving human participants were reviewed and approved by the University Hospital of Larissa Ethics Committee (Protocol number: 13434 and date of approval: 30/03/2022). Written informed consent for participation was not required for this study in accordance with the national legislation and the institutional requirements.

## Author contributions

EK and EZ contributed to conception and design of the study, performed the statistical analysis, and wrote the first draft of the manuscript. GG and NN gathered data. All authors contributed to manuscript revision, read, and approved the submitted version.

## Conflict of interest

The authors declare that the research was conducted in the absence of any commercial or financial relationships that could be construed as a potential conflict of interest.

## Publisher’s note

All claims expressed in this article are solely those of the authors and do not necessarily represent those of their affiliated organizations, or those of the publisher, the editors and the reviewers. Any product that may be evaluated in this article, or claim that may be made by its manufacturer, is not guaranteed or endorsed by the publisher.
